# 2-Year BMI Changes of Children Referred for Multidisciplinary Weight Management

**DOI:** 10.1155/2014/152586

**Published:** 2014-01-30

**Authors:** Jennifer K. Cheng, Xiaozhong Wen, Kristen D. Coletti, Joanne E. Cox, Elsie M. Taveras

**Affiliations:** ^1^Division of General Pediatrics, Department of Medicine, Boston Children's Hospital, Boston, MA 02115, USA; ^2^Division of Behavioral Medicine, Department of Pediatrics, School of Medicine and Biomedical Sciences, State University of New York at Buffalo, Buffalo, NY 14214, USA; ^3^Obesity Prevention Program, Department of Population Medicine, Harvard Medical School and Harvard Pilgrim Health Care Institute, Boston, MA 02215, USA; ^4^Division of General Pediatrics, Massachusetts General Hospital, Boston, MA 02114, USA

## Abstract

*Objective.* To examine body mass index (BMI) changes among pediatric multidisciplinary weight management participants and nonparticipants. *Design.* In this retrospective database analysis, we used multivariable mixed effect models to compare 2-year BMI *z*-score trajectories among 583 eligible overweight or obese children referred to the One Step Ahead program at the Boston Children's Primary Care Center between 2003 and 2009. *Results.* Of the referred children, 338 (58%) attended the program; 245 (42%) did not participate and were instead followed by their primary care providers within the group practice. The mean BMI *z*-score of program participants decreased modestly over a 2-year period and was lower than that of nonparticipants. The group-level difference in the rate of change in BMI *z*-score between participants and nonparticipants was statistically significant for 0–6 months (*P* = 0.001) and 19–24 months (*P* = 0.008); it was marginally significant for 13–18 months (*P* = 0.051) after referral. Younger participants (<5 years) had better outcomes across all time periods examined. *Conclusion.* Children attending a multidisciplinary program experienced greater BMI *z*-score reductions compared with usual primary care in a real world practice; younger participants had significantly better outcomes. Future research should consider early intervention and cost-effectiveness analyses.

## 1. Introduction

Pediatric obesity is a serious health condition, conferring both immediate and long-term health risks [1–3]. Multidisciplinary approaches in diverse sectors, including pediatric primary care, have been proposed to reduce the high prevalence of childhood obesity [4].

Multi-disciplinary clinical programs require a considerable investment of time and resources, but limited data exists on long-term weight outcomes of children participating in such programs, and few studies have examined real-world pediatric weight management of different intensity.

The purpose of this study was to examine changes in body mass index (BMI) among children who were referred to a multi-disciplinary weight management program. We were particularly interested in learning whether there were differences in weight outcomes among program participants as compared with nonparticipants who continued to be followed by their primary care providers within the group practice over a 2-year period following referral. We designated program participants as the “intervention group” and non-participants as the “comparison group” for the purpose of this study, although we recognized that this analysis was based on observational rather than experimental data.

## 2. Methods

### 2.1. Setting and Study Design

The One Step Ahead (OSA) program was developed in 2003 specifically to provide stepped-up care for the growing numbers of children with increasing obesity severity within the Boston Children's Primary Care Center (CHPCC). The CHPCC practice comprises over 80 healthcare providers annually serving more than 14,000 children from mostly economically challenged neighborhoods in Boston, MA. Approximately 44% of children seen for well-child care are overweight or obese; 65% are insured through Medicaid.

In this retrospective observational study, we used the clinically derived OSA database to compare BMI *z*-score changes among program participants (intervention group) versus non-participating children who were followed by primary care providers within the group practice (comparison). The study protocol was approved by the human subjects committee of Boston Children's Hospital.

### 2.2. Study Population

The study population comprised overweight (BMI ≥ 85th and <95th sex- and age-specific percentile based on Centers for Disease Prevention and Control 2000 Growth Charts) or obese (>95th percentile) children aged 2–18 years who were referred from the CHPCC to the OSA program between 2003 and 2009. The *intervention group* comprised children who attended the OSA program and had completed at least 2 visits of any type where BMI was measured in the 2-year period following referral. The* comparison group* comprised referred children who did not keep their OSA appointments, but had nonetheless completed at least 2 primary care visits where BMI was measured over the same 2-year period.

### 2.3. Intervention

The OSA team includes medical providers, a nurse educator, registered dietitians, a behavioral psychologist, a social worker, and a physical activity coordinator. The goal of the program is to achieve weight maintenance or loss among children as they continue growing in height. The OSA program utilizes the social ecological model as a framework for its services and considers individual, interpersonal, community, and societal levels of influences on the child's behavior change. Motivational interviewing [5, 6] techniques are used to assess families' readiness to change, help families set achievable healthful lifestyle goals, and navigate potential obstacles.

Dieticians provide family-centered nutrition education and teach families practical skills including meal planning, label reading, and culturally appropriate healthful cooking techniques. A physical activity coordinator matches families with free or low-cost neighborhood exercise programs. A behavioral psychologist evaluates families for maladaptive behaviors and provides supportive mental health services including individual and family counseling to bolster self-esteem and resiliency. A medical social worker frequently assists families with acute social support needs including housing, transportation, utilities, and food assistance. In general, visits are scheduled at monthly intervals for at least the first 3 visits and for a total of 6 visits over the course of 12 months; however, actual visit intervals and total program duration are quite variable due to the highly individualized nature of the program. The OSA program staff calls the families of children who do not keep their OSA appointments to help them reschedule missed visits and emails referring providers to inform them about the missed visit. Approximately 40% of families are reached by phone and the most common reasons given for missing their scheduled OSA visits include forgetting about the appointment, transportation issues, and scheduling conflicts with work, school or other competing priorities. Many of the families served by the OSA clinic struggle with social stressors including unemployment, food, or home insecurity, and may have difficulty affording basic needs such as clothing, electricity, or telephone service.

Children who were referred but did not keep any OSA appointments were seen by their primary care providers for routine well child-care annually or more frequently for problem-focused visits (e.g., for weight related or other issues). CHPCC well care visit content is based on the *Bright-Futures Guidelines for Health Supervision* [7], but no practice-wide standards existed at the time of this study for obesity assessment, education or follow-up intervals. CHPCC providers manage obese children using general patient education, materials. Among 245 referred children who did not attend the OSA program, 17.3% had weight monitoring visits in addition to well child-care visits with their primary care providers.

### 2.4. Outcomes

At each visit, clinical assistants measured the child's weight and height. We calculated BMI as “weight in kg/height in meters^2^” and then used the Centers for Disease Prevention and Control (CDC) 2000 Growth Charts [8] to calculate gender- and age-specific BMI *z*-score. Change in BMI *z*-score at each postreferral visit was calculated as current BMI *z*-score minus baseline BMI *z*-score at referral. The primary outcome was the rate of change in BMI *z*-score (defined as “difference in BMI z-score/time interval”) per month during each of 4 time periods within 24 months after referral: referral-6 months, 7–12 months, 13–18 months, and 19–24 months.

Covariates of interest included gender, age at referral, race/ethnicity (Black, White, Hispanics, and other), language (English, Spanish, and other), and baseline BMI *z*-score at referral.

### 2.5. Statistical Analysis

To examine group differences in baseline characteristics, we performed Chi-square tests for categorical variables (e.g., gender) and *t*-tests for continuous variables (e.g., age).

Because each child in the analytic sample had multiple visits and childhood BMI usually tracks with age, we used mixed effect models to examine the rate of change in BMI *z*-score after referral. In our sample, an autoregressive correlation structure was chosen for different visits of the same child in the final model, because this structure was associated with a lower Akaike Information Criterion than several other candidate correlation structures. Briefly, an auto-regressive correlation structure indicates that two BMI *z*-scores observed at two closer visits for a particular child tend to be more correlated than two BMI *z*-scores observed at farther apart visits. Specifically, we specified a random effect for the intercept reflecting between-subject variation in baseline BMI *z*-score at referral, and specified a fixed effect for intervention, time after referral and potential interaction between intervention and time after referral. We used piece-wise linear methods to allow for nonlinear trends of change in BMI *z*-score. Specifically, we first divided the 2 years of followup into the four 6-month periods (i.e., referral-6 months, 7–12 months, 13–18 months, and 19–24 months) and then considered a linear trend within each period. Accordingly, we fit a series of hierarchical models with main effects of intervention and time; 2-way interaction between Intervention and time; and 3-way interactions between intervention, time, and the 4 time periods. For the purpose of visualization, we used the smoothing function in Microsoft Excel to connect the 4 time periods smoothly and then to compare the group-level mean BMI *z*-score trajectories between the intervention and comparison groups (Figure 2).

All regression models were adjusted for potential confounders: the child's gender, age at referral, race/ethnicity, primary language, and BMI *z*-score at referral. We also tested for potential interactions between intervention group and demographic characteristics by performing stratified analyses by the child's gender, race/ethnicity, age at referral, and primary language.

We conducted all analyses in SAS 9.2 (SAS Institute Inc., Cary, NC).

## 3. Results

### 3.1. Comparison of Baseline Characteristics

Table 1 shows the characteristics of the multi-disciplinary intervention (OSA, *N* = 338) and comparison (*N* = 245) groups. The mean (standard deviation or SD) age of patients at the time of referral to the OSA program was 8.7 (2.6) years, mean BMI *z*-score was 2.3 (0.5) units, and the prevalence of obesity was 93.5%. The two groups did not differ by gender, age, race, BMI, or prevalence of obesity. However, children in the intervention group had a slightly higher mean BMI *z*-score (2.3 versus 2.2), more visits within 2 years after referral (mean number of visits, 5.5 [3.2] versus 2.5 [1.2]), and were more likely to speak Spanish as a primary language (21.0% versus 14.3%). Most of the OSA team providers including all physicians also speak Spanish.

### 3.2. Distribution of Visits

**Figure 1:**
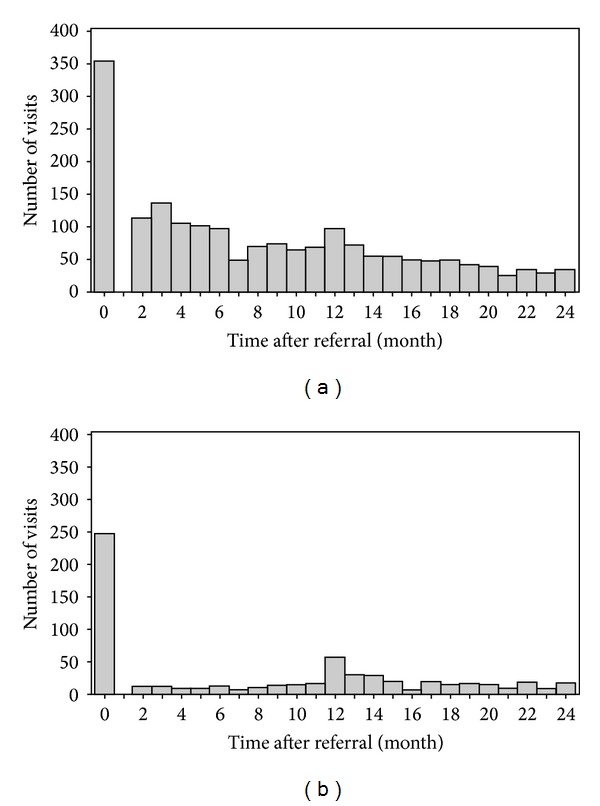
Distribution of time at visits for OSA and comparison groups: (a) OSA group (1,855 visits by 338 children); (b) comparison group (615 visits by 245 children).

Figure 1 shows the distribution of visits for the 2 groups. There were a total of 1,855 visits for the intervention group and 615 visits for the comparison group. For both groups, the visits after referral dispersed between 2 months and 24 months. The visit frequency decreased over time in the OSA group. The mean time for follow-up visits was similar between the two groups (8.4 versus 8.1 months after referral). As shown in Table 1, 855 (26%) children in the OSA group and 43 (17.6%) children in the comparison group were followed to 2 years (22–24 months). The mean follow-up time was slightly longer for the OSA group than the comparison group (14.8 (SD, 7.2) versus 13.3 (SD, 7.7) months after referral; *P* = 0.01).

### 3.3. Change in BMI *z*-Score after Referral

**Figure 2:**
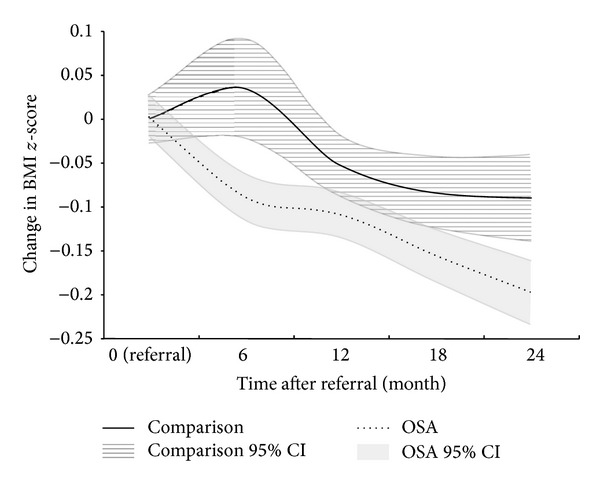
Change in BMI *z*-score after referral for OSA and comparison groups.

Figure 2 shows change in BMI *z*-score at 6, 12, 18, and 24 months after referral. For the intervention group, the mean BMI *z*-score decreased more steeply during the first 6 months and continuously during the 2-year period. In contrast, the mean BMI *z*-score for the comparison group increased during the first 6-postreferral months then decreased to 18 months, after which it leveled off.

Table 2 shows the estimated rates of change in BMI *z*-score (units per month) for the 4 time periods. From 0 to 6 months, BMI *z*-score decreased for the intervention group at the rate of −0.013 units/month (95% confidence interval or CI, −0.017 to −0.009)) but increased for the comparison group (0.004 units/month (95% CI, −0.005 to 0.013)). The BMI *z*-score continued to decrease more steeply for the intervention group than the comparison group from 7 to 12 months (−0.008 versus −0.005), 13 to 18 months (−0.008 versus −0.005), and 19 to 24 months (−0.008 versus −0.004). The group difference (interventional versus comparison) in the rate of change in BMI *z*-score was statistically significant for 0–6 months (*P* = 0.001) and 19–24 months (*P* = 0.008); it was marginally significant for 13–18 months (*P* = 0.051). Among OSA participants, there were no significant racial/ethnic differences except non-Hispanic Black children who had a smaller decrease in BMI *z*-score than all other groups during the 0 to 6-month period (−0.007 versus −0.017, *P* = 0.009). There were also no significant group-differences in the rate of change in BMI *z*-score based on gender. Among OSA participants, children younger than 5 years had significantly greater decreases in BMI *z*-score than older children during all time periods (0 to 6 months: −0.067 versus −0.005, *P* = 0.001; 7 to 12 months: −0.032 versus −0.003, *P* = 0.001; 13 to 18 months, −0.024 versus −0.004, *P* = 0.001; 19 to 24 months, −0.024 versus −0.004, *P* value = 0.001).

## 4. Discussion

In this study, we found that a group of racial ethnically diverse children attending a multi-disciplinary weight management program experienced reductions in BMI *z*-score over a 2-year follow-up period. These BMI reductions were of greater magnitude than those of non-participants but were overall modest. Among OSA program participants, younger children experienced significantly greater reductions in BMI *z*-score than older children, but we did not observe gender or racial/ethnic differences. Our findings add to the scarce evidence on the effectiveness of real-world pediatric weight management among overweight children of diverse races/ethnicities and low family socioeconomic status.

The reasons for the modest reduction in BMI *z*-score seen among OSA participants are likely multifactorial. A large proportion of our families have endorsed challenging ongoing psychosocial stressors including limited financial resources to purchase healthful items or enroll children in organized physical activity programs, food or housing insecurity, competing priorities, simultaneous exacerbations of chronic illness in a family member, and difficulty sustaining motivation over time as significant barriers to maintaining a healthy weight. In addition, a number of biological adaptations including changes in the circulating levels of several peripheral hormones involved in the homeostatic regulation of body weight likely contribute to weight gain recidivism. Sumithran et al. found that circulating levels of leptin, peptide YY, cholecystokinin, insulin, ghrelin, gastric inhibitory polypeptide, and pancreatic polypeptide, as well as subjective feelings of hunger, do not revert to the preweight loss levels even one year after initial weight reduction [9]. While the BMI changes we observed were modest, a necessary level (threshold) of pediatric BMI change associated with health benefits among children has not been established. Adult studies have shown that even modest weight loss (5–10%) is associated with cardiovascular benefits [10]. Among children, some experts have suggested that even BMI stabilization/ maintenance might be considered to be a successful endpoint [11], especially given the almost linear increase in BMI after adiposity rebound at age 5-6 y experienced by most children [12]. In addition, psychological benefits associated with even modest weight loss (and the lifestyle changes accompanying such weight loss) among children include a sense of mastery and improved self-esteem[13].

While children and their parents choosing to attend our weight management programs might inherently possess higher motivation relative to nonattendees, our findings provide invaluable insight as a reflection of “real-world” clinical practice as randomization is not always practical in service priority settings. The One Step Ahead program was developed specifically to meet demands for readily accessible, culturally sensitive, multidisciplinary weight management services for the growing numbers of obese children in our safety-net practice serving families predominantly from low-income, racial ethnically diverse Boston neighborhoods. Embedded within a busy academic primary care practice of more than 80 pediatricians in cohabitation with several other clinical programs, OSA program research capacity is limited by space, scheduling, staffing, and budgetary constraints and subsumed by the impetus to deliver patient-centered care in a medical home practice model. Our approach represents a realistic, culturally appropriate weight management intervention targeting a predominantly low-income Hispanic or African-American population and may inform many similar programs where randomization may not be feasible, acceptable, or sustainable on a long-term basis.

Limited evidence has suggested the greater effectiveness of medium- to high-intensity behavioral interventions, compared with low-intensity interventions conducted in primary care settings [14]. The Expert Committee Recommendations for Childhood Obesity Management [15] propose a staged approach to obesity treatment. Stage 1, “Prevention Plus,” comprises brief counseling regarding key healthful lifestyle behaviors that can be delivered in primary care office settings; Stage 2, “Structured Weight Management,” delivers similar messages through the added structure of a dietician or other trained professional such as an exercise counselor. Stage 3, “Comprehensive Multidisciplinary Intervention,” is a structured program in behavior modification that includes goal setting and contingency management facilitated by a team approach. Stage 4, “Tertiary Care Intervention” includes the use of medications and bariatric surgery as potential treatment modalities and is reserved for children who are refractory to more conservative treatment at the lower stages. Children in the One Step Ahead program can be considered Stage 3 treatment recipients, while those in the comparison group (referred but did participate and were followed by primary care physicians) can be considered Stage 1 treatment recipients. The steadier and greater reduction in BMI *z*-score seen with children enrolled in our “Stage 3” intervention compared with children in the comparison “Stage 1” strategy appears to support the premise of higher effectiveness for more intensive approaches. If more intensive treatment more effectively reduces BMI, a staged treatment approach per the Expert Committee Guidelines seems sensible. However, we do not contend that greater decreases in BMI alone result in better outcomes in the pediatric population. It may also be important to account for the impact of nonweight outcomes on child and family well being. In the era of Accountable Care Organizations [16], for the greater resources necessary per unit increased BMI effect, whether children should be treated with Stage 3 and Stage 4 interventions at all requires further study to first define and then maximize core health outcomes for the resources expended.

Careful examination of the BMI *z*-score trajectory over time can offer additional insights regarding why our intervention works and how to improve it in future. First, the overall decline in BMI *z*-scores observed over the 2-year study period among intervention participants suggests that weight management may be maintained over time, although the effect size may decrease. This is encouraging given the recidivism of weight gain due to compensatory metabolic processes that resist the maintenance of the altered body weight [17]. The rise in BMI *z*-scores during the initial 0–6-month period among comparison children may be related to delayed followup by providers who had referred the patients to OSA, only to find later that the families had not participated. Secondly, the greatest difference in the change in BMI *z*-score between intervention (decrease) and comparison (increase) children occurred in the 0–6 months period. This difference may be related to higher initial self-motivation and vigilance, more intensive intervention within the earlier periods, or higher-impact behavioral changes that represent “low-lying fruit,” where later improvements may be more incremental and require greater efforts. These findings are consistent with previous work showing greater intervention impact on weight outcomes in the early periods of weight management [18]. Interestingly, BMI *z*-scores for the comparison group also decreased following the initial rise, although being at a lesser rate than for the intervention group, suggesting that the Stage 1 strategy by primary care providers in our CHPCC is also somewhat effective. Therefore, closer followup by primary care providers is needed to ensure that referral or primary provider care occurs. Standardized time intervals for followup of obese children could potentially improve outcomes.

Among children who attended our multidisciplinary program, we found that younger age was associated with better weight outcomes, which is consistent with a growing body of evidence linking earlier intervention with better long-term weight outcomes. For example, in two long-term follow-up studies of randomized trials, Brotman et al. found that children at risk for behavioral problems who received a family intervention to promote effective parenting at age 4 y had lower BMI and improved health behaviors in preadolescence [19]. Reinehr et al. found that younger age (<8 years) predicted the best long-term weight outcomes among obese children enrolled in a year-long lifestyle intervention [20]. These findings collectively support the premise that early prevention may optimize weight outcomes in high-risk children, with important implications for future childhood obesity interventions.

### 4.1. Limitations

Our study is limited by its retrospective observational design, which did not allow for the allocation of subjects in a randomized-controlled fashion to control unmeasured confounders such as self-motivation of changing lifestyle. However, a stratified data analysis by gender, race/ethnicity, and primary language did not yield substantial differences in estimated intervention effects, so it is unlikely that any unobserved allocation imbalances of baseline characteristics could completely explained the significant group differences in BMI *z*-score we observed. Other limitations include selection bias due to eligibility criteria or loss to follow up since families with higher initial self-motivation and vigilance were more likely to accept and/or continue the intervention; variations in intervention activities, intensity, and length; and residual confounding by family socioeconomics. Finally, the primary-care embedded within a tertiary hospital setting model may limit its generalizability.

## 5. Conclusion

In this study, a group of racial-ethnically diverse overweight children attending a multidisciplinary weight management program demonstrated sustained but modest BMI *z*-score reductions over a 2-year period. Younger children (<5 years) had significantly better weight outcomes compared with older children. Our findings suggest that multi-disciplinary programs might be considered as a treatment option within the spectrum of pediatric obesity management in health care settings and also that interventions targeting younger children might have greater impact. It is our next step to refine our intervention strategies to further increase their effectiveness. Finally, future studies ought to evaluate the promise of early intervention and also consider cost-effectiveness analyses.

## Figures and Tables

**Table 1:** Characteristics and visit frequency of analytic samples for the OSA and comparison groups.

	OSA	Comparison	*P* value
*Child level *			
Total number of children	338	245	
Gender, %			
Boys	46.5	49.0	0.55
Girls	53.6	51.0	
Age at referral, %			
2–5 y	11.0	13.9	
6–8 y	35.5	25.3	
9–10 y	27.2	33.1	
11–18 y	26.3	27.8	
Mean (SD)	8.7 (2.6)	8.8 (2.8)	0.47
Race, %			
White	5.6	3.3	0.10
Black	51.8	61.6	
Hispanic	24.0	18.8	
Others	18.6	16.3	
Language, %			
English	74.3	84.1	0.01
Spanish	21.0	14.3	
Others	4.7	1.6	
Prevalence of obesity at baseline, %	93.5	91.0	0.27
Baseline BMI, mean (SD)	26.5 (3.9)	26.2 (4.6)	0.37
Baseline BMI *z*-score, mean (SD)	2.3 (0.5)	2.2 (0.5)	0.03
Number of all-type visits, mean (SD)	5.5 (3.2)	2.5 (1.2)	<0.001
Number of OSA visits, mean (SD)	2.7 (1.9)	N/A	—
Duration of OSA intervention in months, mean (SD)	9.5 (6.9)	N/A	—
Time after referral at the last visit (months)			
0–6 months	21.2	19.2	
7–12 months	11.0	11.8	
13–15 months	24.9	15.4	
16–18 months	11.4	11.0	
19–21 months	13.9	16.6	
22–24 months	17.6	26.0	
Mean (SD)	14.8 (7.2)	13.3 (7.7)	0.01
*Visit level *			
Total number of visits	1855	615	
Time after referral in months, mean (SD)	8.4 (7.0)	8.1 (8.0)	0.49

**Table 2:** Rate of change in BMI *z*-score (units per month) after referral for OSA and comparison groups.

Time period	Mean rate of change in BMI *z*-score (95% CI)*			*P* value
	OSA group	Comparison group	Mean difference (OSA-comparison)	
Referral-6 m	−0.013 (−0.017, −0.009)	0.004 (−0.005, 0.013)	−0.017 (−0.027, −0.007)	0.001
7–12 m	−0.008 (−0.011, −0.006)	−0.005 (−0.008, −0.003)	−0.003 (−0.007, 0.001)	0.093
13–18 m	−0.008 (−0.010, −0.006)	−0.005 (−0.007, −0.003)	−0.003 (−0.006, 0.000)	0.051
19–24 m	−0.008 (−0.010, −0.006)	−0.004 (−0.006, −0.002)	−0.004 (−0.006, −0.001)	0.008

*Adjusted for the child's gender, age at referral, race/ethnicity, spoken language, and BMI *z*-score at referral.
